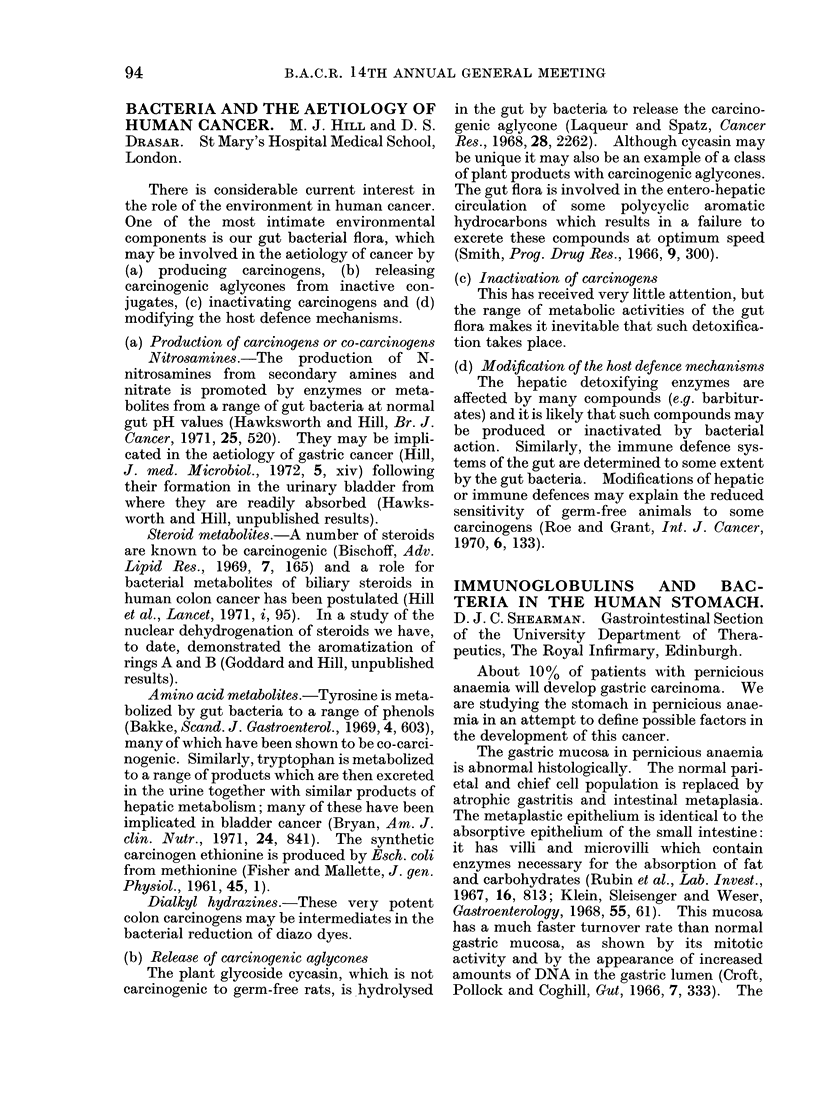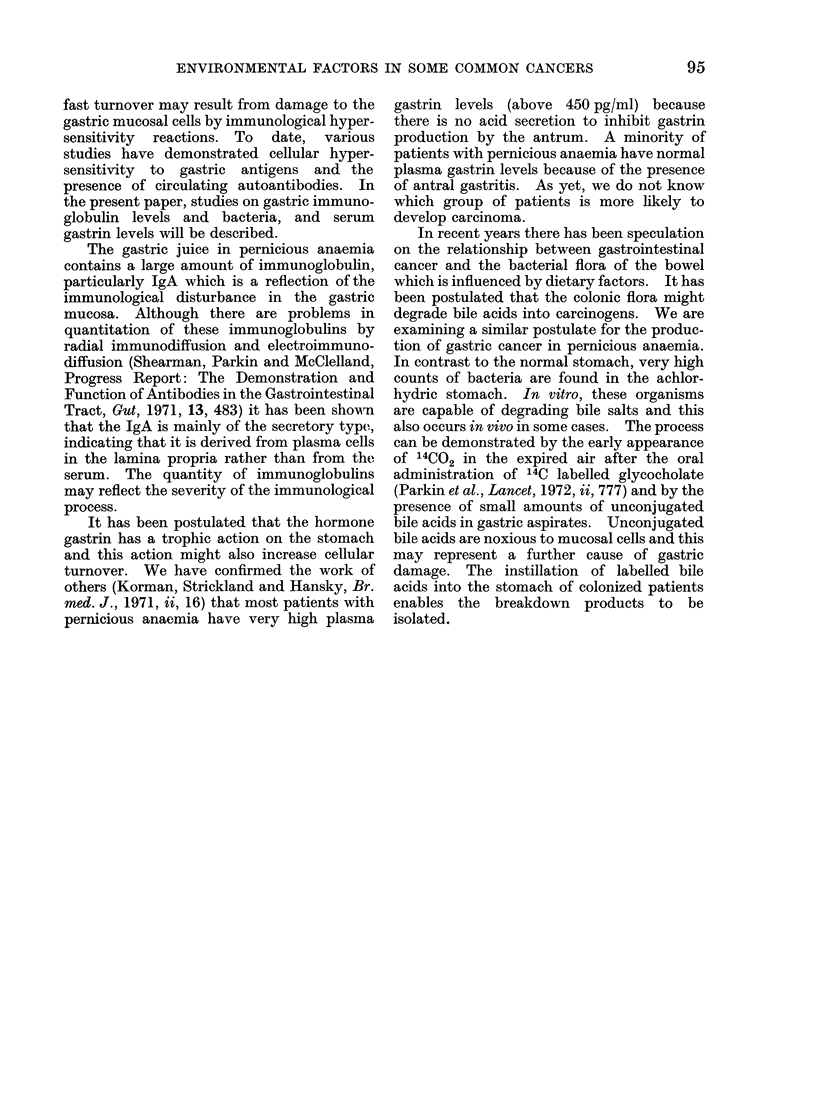# Immunoglobulins and bacteria in the human stomach.

**DOI:** 10.1038/bjc.1973.123

**Published:** 1973-07

**Authors:** D. J. Shearman


### IMMUNOGLOBULINS AND BACTERIA IN THE HUMAN STOMACH.

D. J. C. SHEARMAN. Gastrointestinal Section of the University Department of Therapeutics, The Royal Infirmary, Edinburgh.

About 10% of patients with pernicious anaemia will develop gastric carcinoma. We are studying the stomach in pernicious anaemia in an attempt to define possible factors in the development of this cancer.

The gastric mucosa in pernicious anaemia is abnormal histologically. The normal parietal and chief cell population is replaced by atrophic gastritis and intestinal metaplasia. The metaplastic epithelium is identical to the absorptive epithelium of the small intestine: it has villi and microvilli which contain enzymes necessary for the absorption of fat and carbohydrates (Rubin *et al.*, *Lab. Invest.*, 1967, **16**, 813; Klein, Sleisenger and Weser, *Gastroenterology*, 1968, **55**, 61). This mucosa has a much faster turnover rate than normal gastric mucosa, as shown by its mitotic activity and by the appearance of increased amounts of DNA in the gastric lumen (Croft, Pollock and Coghill, *Gut*, 1966, **7**, 333). The fast turnover may result from damage to the gastric mucosal cells by immunological hypersensitivity reactions. To date, various studies have demonstrated cellular hypersensitivity to gastric antigens and the presence of circulating autoantibodies. In the present paper, studies on gastric immunoglobulin levels and bacteria, and serum gastrin levels will be described.

The gastric juice in pernicious anaemia contains a large amount of immunoglobulin, particularly IgA which is a reflection of the immunological disturbance in the gastric mucosa. Although there are problems in quantitation of these immunoglobulins by radial immunodiffusion and electroimmunodiffusion (Shearman, Parkin and McClelland, Progress Report: The Demonstration and Function of Antibodies in the Gastrointestinal Tract, *Gut*, 1971, **13**, 483) it has been shown that the IgA is mainly of the secretory type, indicating that it is derived from plasma cells in the lamina propria rather than from the serum. The quantity of immunoglobulins may reflect the severity of the immunological process.

It has been postulated that the hormone gastrin has a trophic action on the stomach and this action might also increase cellular turnover. We have confirmed the work of others (Korman, Strickland and Hansky, *Br. med. J.*, 1971, *ii*, 16) that most patients with pernicious anaemia have very high plasma gastrin levels (above 450 pg/ml) because there is no acid secretion to inhibit gastrin production by the antrum. A minority of patients with pernicious anaemia have normal plasma gastrin levels because of the presence of antral gastritis. As yet, we do not know which group of patients is more likely to develop carcinoma.

In recent years there has been speculation on the relationship between gastrointestinal cancer and the bacterial flora of the bowel which is influenced by dietary factors. It has been postulated that the colonic flora might degrade bile acids into carcinogens. We are examining a similar postulate for the production of gastric cancer in pernicious anaemia. In contrast to the normal stomach, very high counts of bacteria are found in the achlorhydric stomach. *In vitro*, these organisms are capable of degrading bile salts and this also occurs *in vivo* in some cases. The process can be demonstrated by the early appearance of $^{14}CO_2$ in the expired air after the oral administration of $^{14}C$ labelled glycocholate (Parkin *et al.*, *Lancet*, 1972, *ii*, 777) and by the presence of small amounts of unconjugated bile acids in gastric aspirates. Unconjugated bile acids are noxious to mucosal cells and this may represent a further cause of gastric damage. The instillation of labelled bile acids into the stomach of colonized patients enables the breakdown products to be isolated.